# How little data is enough? Phase-diagram analysis of sparsity-regularized X-ray computed tomography

**DOI:** 10.1098/rsta.2014.0387

**Published:** 2015-06-13

**Authors:** J. S. Jørgensen, E. Y. Sidky

**Affiliations:** 1 Department of Applied Mathematics and Computer Science, Technical University of Denmark, Richard Petersens Plads, Kongens Lyngby 2800, Denmark; 2 Department of Radiology MC-2026, University of Chicago, 5841 South Maryland Avenue, Chicago, IL 60637, USA

**Keywords:** computed tomography, compressed sensing, image reconstruction, sparsity regularization, sampling

## Abstract

We introduce phase-diagram analysis, a standard tool in compressed sensing (CS), to the X-ray computed tomography (CT) community as a systematic method for determining how few projections suffice for accurate sparsity-regularized reconstruction. In CS, a phase diagram is a convenient way to study and express certain theoretical relations between sparsity and sufficient sampling. We adapt phase-diagram analysis for empirical use in X-ray CT for which the same theoretical results do not hold. We demonstrate in three case studies the potential of phase-diagram analysis for providing quantitative answers to questions of undersampling. First, we demonstrate that there are cases where X-ray CT empirically performs comparably with a near-optimal CS strategy, namely taking measurements with Gaussian sensing matrices. Second, we show that, in contrast to what might have been anticipated, taking randomized CT measurements does not lead to improved performance compared with standard structured sampling patterns. Finally, we show preliminary results of how well phase-diagram analysis can predict the sufficient number of projections for accurately reconstructing a large-scale image of a given sparsity by means of total-variation regularization.

## 1. Introduction

### (a) Sparsity regularization in X-ray CT

Sparsity-regularized (SR) image reconstruction has shown great promise for X-ray computed tomography (CT). Many works, e.g. [1–6], have demonstrated that accurate reconstructions can be obtained from substantially less projection data than is normally required by standard analytical methods such as filtered back-projection and algebraic reconstruction methods. Acquiring less data is of interest in many applications of X-ray CT to reduce scan time or exposure to ionizing radiation.

The typical SR set-up for X-ray CT, and the one we employ, is that an unknown discrete image 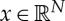 is to be reconstructed from measured discrete data 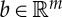, connected to *x* through a linear model, *b*≈*Ax*, for some measurement matrix 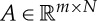. A common reconstruction problem is
1.1
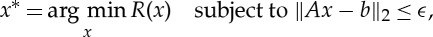

where *R*(*x*) is a sparsity regularizer, for example the 1-norm, the total variation (TV) semi-norm or a 1-norm of wavelet coefficients or coefficients in a learned dictionary, depending on which domain sparsity is expected in, and *ϵ* is a regularization parameter that must be chosen to balance the level of regularization enforced with the misfit to data.

In contrast to analytical and algebraic reconstruction methods, SR can admit reconstructions in the underdetermined case *m*<*N* as shown in the references given above. However, from the existing individual studies, it is difficult to synthesize a coherent quantitative understanding of the undersampling potential of SR in CT. From a practical point of view, we want to know how many CT projections to acquire in order to obtain an SR reconstruction of sufficient quality to reliably solve the relevant imaging task, for example detection, classification, segmentation, etc. This question is difficult to address meaningfully in general, because specific applications pose different challenges, for example varying levels of noise and inconsistencies in the data as well as different quality requirements on the reconstruction. But even in an application-independent setting, systematic analysis of the undersampling potential of SR in CT remains unexplored.

We consider in the present work an idealized form of the reconstruction problem (1.1) with *ϵ*=0 and consider only synthetic noise-free data. This simplified set-up allows us to study more precise questions with fewer complicating factors involved. Specifically, we consider the three reconstruction problems, P_1_, LP and TV:

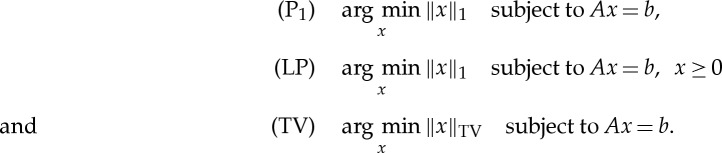

The first two are standard 1-norm minimization (the latter with non-negativity constraint enforced) for reconstruction of images sparse in the image domain. The last is TV minimization for sparsity in the gradient domain. The TV semi-norm is defined as

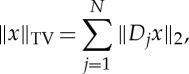

where *D*_*j*_ is a finite-difference approximation of the gradient at pixel *j*. In this work, we use forward differences and Neumann boundary conditions.

In the idealized set-up, we are interested in the central property of *recoverability*: an image is said to be recoverable (from its ideal synthetic data) if it is the unique solution to the considered reconstruction problem. For example, we say that an image *x*_orig_ is recoverable by P_1_ from data *b*=*Ax*_orig_ if *x*_orig_ is the unique P_1_ solution. The fundamental question we are interested in is: How few samples are enough for recovery of an image of a given sparsity by SR reconstruction?

In other words, we want to study recoverability as a function of sparsity and sampling levels. In the present work, we will develop and apply a systematic analysis tool known as phase-diagram analysis from the field of compressed sensing (CS) for this purpose in the setting of CT.

### (b) Compressed sensing

The field of CS addresses precisely the question of how few samples one can acquire and still *provably* recover the image. In general, obviously, we need *N* linearly independent samples of an image 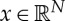 to recover *x*. Central to CS is sparsity: an image is said to be *s*-sparse if it has at most *s* non-zero entries. What CS says is that if the image *x* is sufficiently sparse then by taking the right kind of samples, we can recover *x* by SR from fewer than *N* samples. Furthermore, the more sparse *x* is, the fewer samples will suffice. CS was initiated with the works of Donoho [7] and Candès and co-workers [8,9]. Before the advent of CS, SR reconstruction using the 1-norm had been used heuristically for reduced sampling in CT [10,11], but the works of Donoho and Candès *et al.* sparked renewed interest and a new focus on guarantees of accurate reconstruction.

An important quantity for CS guarantees is the restricted isometry property (RIP), which is defined as follows. A matrix *A* is said to satisfy the RIP of order *s* if there exists a constant *δ*_*s*_∈(0,1) such that for all *s*-sparse signals *x* it holds that
1.2
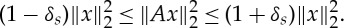

An example of an RIP-based CS guarantee is (e.g. [12]): if a matrix *A* satisfies the RIP with 
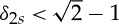
, then all *s*-sparse images *x* will be recovered by P_1_ from data *b*=*Ax*.

The problem is then to identify matrices satisfying this, and unfortunately computing RIP constants is in general NP-hard [13]. An important class of matrices that admit RIP results are the Gaussian sensing matrices, for which matrix elements are independent samples from the zero-mean, unit-variance normal distribution. If the number of measurements *m* satisfies
1.3
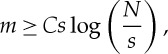

where *C* is a constant, then with high probability a Gaussian sensing matrix possesses the RIP, such that all *s*-sparse images *x* will be recovered.

In a certain sense, the Gaussian sensing matrices constitute a *near-optimal sampling strategy* [12,14], because no other matrix type can provide the same recovery guarantee for fewer samples than (1.3). The importance of the Gaussian sensing matrices in CS is further established by many additional guarantees based, for example, on incoherence of the sensing matrix. It is not our intention to give a comprehensive review of CS theory here; such can be found in many places, for example the recent overview by Foucart & Rauhut [15].

The prominent role of the Gaussian sensing matrices and other random matrix constructions in CS gives the impression that random sensing is a key CS feature and it is tacitly assumed that random sensing provides superior recoverability performance to that of structured sampling. This assumption has even led researchers to investigate hardware implementations of random sampling for CT [16]. However, more recently, novel CS guarantees have appeared for certain *non-random* matrices [17], which may be a step towards reduced focus on random sampling, although these matrices are also quite far from those of CT.

It is generally well understood [15,18] that current CS theory does not cover deterministic sampling set-ups in real-world applications. For CT, in particular, Petra & Schnörr [19,20] showed that CS guarantees are extremely poor. The main sensing problem of CT is its fundamental nature of sampling the object by line integrals. Each line integral only samples a small part of the object, thus leading to sparse, highly structured and coherent CT sampling matrices. By contrast, CS sensing matrices, such as the Gaussian, are dense, have random elements and are incoherent, and hence fundamentally different. In other words, there remains a large gap between the empirically observed effectiveness of SR in CT and the mathematical CS guarantees of accurate recovery typically involving random matrices.

### (c) Own previous work and contribution of present work

We have recently been interested in analysing SR in CT from a CS perspective [21–23]. More specifically, we have studied recoverability from fan-beam CT data by 1-norm and TV regularization. We introduced the use of certain phase diagrams from CS to the setting of CT for systematically studying how recoverability depends on sparsity and sampling. Our work demonstrated quantitatively that recoverability from equi-angular fan-beam CT data for certain classes of test images exhibits a phase-transition phenomenon very similar to what has been proved in CS for the Gaussian sensing matrices, as will be explained in §2.

In the present work, we will further refine the phase-diagram analysis we introduced in [22,23] and demonstrate how it can be used to systematically provide quantitative insight into the undersampling potential of SR in CT by applying it to three cases. First, in §2, we will give the sufficient theoretical background on phase-diagram analysis and the application to CT. Following that, we address in §§3–5 the following studies:

*Study A*. How does CT sampling compare in terms of recoverability to a near-optimal CS sampling strategy, i.e. using Gaussian sensing matrices?
*Study B*. Is recoverability improved by taking random CT measurements?
*Study C*. How accurately can small-scale synthetic-data phase diagrams predict sufficient sampling for realistically sized images of real objects?

Finally, in §6, we conclude the paper.

The purpose of Study A is to put the CT phase-transition behaviour we observed in [22,23] more clearly into context of CS theory. Quite surprisingly, our results demonstrate that standard CT sampling is almost comparable with Gaussian sensing matrices in terms of recoverability. This is surprising since the Gaussian sensing matrices form a near-optimal CS sampling strategy, as explained previously in this section.

Study B addresses the use of random sampling in CT for potentially allowing for accurate reconstruction from fewer measurements than regular structured CT sampling. By use of phase-diagram analysis, we will show that random sampling does *not* lead to improved performance, but rather unchanged or in some cases even substantially reduced performance.

The purpose of Study C is to establish a connection to real-world CT image reconstruction by investigating the practical utility of phase diagrams for predicting how much CT data to acquire for reconstructing accurately a large-scale image of a given sparsity.

In all three studies, we use phase-diagram analysis as the main tool. Our goal is both to arrive at the particular insights of the three studies and to demonstrate phase-diagram analysis as a useful tool for systematically gaining quantitative understanding of SR in CT.

## 2. Phase-diagram analysis

### (a) Theoretical phase-transition results

As explained in §1b, the Gaussian sensing matrices play a central role in CS. It is also possible to give a theoretical description of its P_1_ and LP recoverability in terms of phase-diagram analysis. We present two different theoretical analyses, by Donoho and Tanner (DT) and by Amelunxen, Lotz, McCoy and Tropp (ALMT).

**Figure 1.**
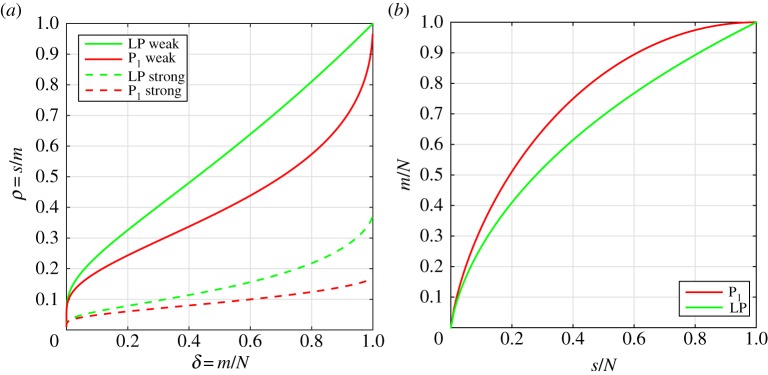
Theoretical phase-transition curves for Gaussian sensing matrices. (*a*) DT asymptotic phase-transition curves for strong and weak recovery by P_1_ and LP; recovery occurs *below* the curves. (*b*) ALMT phase-transition curves for recovery by P_1_ and LP; recovery *above* the curves.

DT established in a series of papers [24–28] phase-transition behaviour of the Gaussian sensing matrices. Their analysis is based on so-called neighbourliness of random polytopes and builds on earlier work by Vershik & Sporyshev [29]. For an *s*-sparse signal 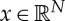 and *m* samples, the DT phase diagram displays recoverability as a function of (*δ*,*ρ*) for *δ*=*m*/*N*∈[0,1] and *ρ*=*s*/*m*∈[0,1]. For the set of *s*-sparse signals, DT consider two notions of recoverability: strong, meaning that *all* *s*-sparse signals are recovered, and weak, meaning that *most* *s*-sparse signals are recovered at a given sampling level. DT then showed for the Gaussian sensing matrices and P_1_ and LP that asymptotically there exist strong/weak phase-transition curves *ρ*(*δ*) such that at a sampling level of *δ* with high probability all/almost all signals with *ρ*<*ρ*(*δ*) will be recovered. Similarly, with high probability not all/almost no signals with *ρ*>*ρ*(*δ*) will be recovered. The strong and weak phase-transition curves for P_1_ and LP are shown in figure 1*a*, plotted from tabulated phase-transition values 30. Below the phase-transition curves are strong and weak full-recovery regions; above the weak phase-transition curves are in addition weak no-recovery regions. We note that the weak full-recovery regions are substantially larger than their strong counterparts and that LP has a larger full-recovery region than P_1_. Both observations intuitively make sense. As we will demonstrate in §3, the asymptotic weak phase-transition curves are in excellent agreement with empirical phase diagrams for finite-sized problems.

ALMT use a completely different analysis [31] based on the so-called statistical dimension of descent cones to prove asymptotic phase-transition behaviour for the Gaussian sensing matrices. The ALMT phase diagram shows recoverability as a function of (*s*/*N*,*m*/*N*)∈[0,1]^2^. ALMT give phase-transition curves, i.e. critical sampling values *m*/*N* as a function of sparsity values *s*/*N* such that most images of a given sparsity are recovered from more samples than the critical level and not recovered from fewer samples. The P_1_ and LP ALMT phase-transition curves are shown in figure 1*b*, computed using the software SNOWMAKER 32. Contrary to the DT phase-transition curves, the full recovery regions are above the curves. We will demonstrate in §3 that the ALMT phase-transition curves are in excellent agreement with empirical phase diagrams.

Regarding recovery guarantees for TV, we are only aware of the RIP results by Needell & Ward [33,34]. To our knowledge, it is an open question whether theoretical phase-transition results can be obtained. In the present work, we demonstrate empirically that such behaviour can be observed from both Gaussian and fan-beam CT sensing matrices.

In addition to the Gaussian sensing matrices, phase-transition behaviour has been observed [25] for several other classes of random matrices and some theoretical analysis has been given [35]. However, it remains open to establish phase-transition behaviour for matrices occurring in practical imaging applications such as CT. Our motivation for the present work is precisely to establish that at least empirically it is possible to observe phase-transition behaviour in CT.

### (b) Experimental procedure of empirical phase-diagram analysis

Even though no theoretical phase-transition results exist for CT, we can construct empirical phase diagrams by repeatedly solving the same reconstruction problem over an ensemble of problem realizations for a range of sparsity and sampling levels. In our case, we found that 100 realizations at each sparsity and sampling level were enough to demonstrate phase-transition behaviour.

Each problem realization is generated in the following way. Given sparsity and sampling levels, a test image *x*_orig_ is generated, a sampling matrix *A* is set up and ideal data *b*=*Ax*_orig_ is computed. From the data *b*, the appropriate reconstruction problem is solved and the reconstruction is denoted x*. Recovery is declared if x* is sufficiently close numerically to *x*_orig_; here, we test whether the relative 2-norm error ∥x*−*x*_orig_∥_2_/∥*x*_orig_∥_2_<*ϵ*, for some choice of threshold *ϵ*. For P_1_ and LP, we found *ϵ*=10^−4^ to be suitable, while for TV we use *ϵ*=10^−3^, as the conic optimization problem is more difficult to solve accurately.

As in [22,23], we use the commercial optimization software MOSEK [36] to solve the reconstruction problems required to construct a phase diagram. MOSEK uses a state-of-the-art primal-dual interior-point method, which allows us to solve P_1_ and LP (recast as linear programs) and TV (recast as a conic program) very accurately. An accurate solution is necessary for correctly assessing numerically whether an image is recovered, as numerical inaccuracies and approximate solutions may lead to the wrong decision. While allowing for high accuracy, interior-point methods are not efficient for large-scale problems. For the reconstruction problems in Study C, we use a large-scale optimization algorithm, which will be described there.

For the Gaussian sensing matrices, each problem realization contains a new realization of the sampling matrix, while in the fan-beam CT case a single matrix (at each sampling level) is used throughout. This is because, in CT, we really are interested in the performance of a fixed matrix, which is specified by the physical scanner geometry.

For the ALMT phase diagrams, we use 39 relative sparsity levels *s*/*N*=0.025,0.050,…,0.975 and 26 sampling levels, namely from 1 to 26 equi-angular projection views. At 26 views, the matrix has size 3338×3228 and is full rank, such that any image, independent of sparsity, will be recovered. For the DT phase diagram, we use the same 26 sampling levels in combination with 32 sparsity levels (relative to the sampling level), i.e. 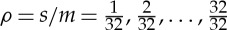.

With 100 realizations at each sparsity and sampling level, a total of 101 400 reconstruction problems need to be solved for a single ALMT phase diagram (at the chosen resolution), while the same number for a DT phase diagram is 83 200. Even with the small images used in this paper, our results have taken many hours of computing time on a cluster at the DTU Computing Center.

## 3. Study A. How does CT compare to compressed sensing?

As we have explained, the Gaussian sensing matrices are central to CS, as they admit strong theoretical results and are shown to form a near-optimal sampling strategy. In this study, we use phase-diagram analysis to compare recoverability of fan-beam CT with the Gaussian sensing matrices. We will show that, despite the lack of CS guarantees for fan-beam CT, we can empirically observe almost comparable recoverability.

### (a) Measurement matrices

We consider two types of measurement matrices: the Gaussian sensing matrices and a system matrix corresponding to a two-dimensional equi-angular fan-beam scanning geometry. A Gaussian sensing matrix is generated by drawing independent, identically distributed elements from the standard zero-mean unit-variance normal distribution.

The two-dimensional fan-beam CT system matrix is practically the same one we used in [22,23], where it is described in detail, and the non-zero structure and the scanning geometry are illustrated in [23]. In brief, we consider a disc-shaped image of *N* pixels in total, inscribed in an *N*_side_×*N*_side_ square pixel array. Fan-beam projections are recorded at *N*_v_ equi-angular views of a 360° scanning arc, each consisting of 2*N*_side_ pixels on a curved detector. The total number of measurements is *m*=*N*_v_⋅2*N*_side_, and the *m*×*N* system matrix is computed by the function fanbeamtomo from the MATLAB^®^ toolbox AIR Tools [37]. The only difference from [22,23] is that the first angle is not chosen to be on a coordinate axes but offset by 20°. This offset regularizes the matrix by avoiding identical rows arising from rays in opposite views aligned with the coordinate axes.

### (b) Image-domain sparsity

#### (i) Signedspikes by P_1_

We consider first the unconstrained problem P_1_. The standard image class considered in CS phase-diagram studies consists of images with random-valued pixels at random locations. We refer to this image class as signedspikes; see [22] for details and illustration. Specifically, we generate a signedspikes image realization as follows: given an image size (number of pixels) *N* and sparsity (number of non-zero pixels) *s*, select uniformly at random *s* pixels and assign values sampled from the uniform distribution on [−1,1].

**Figure 2.**
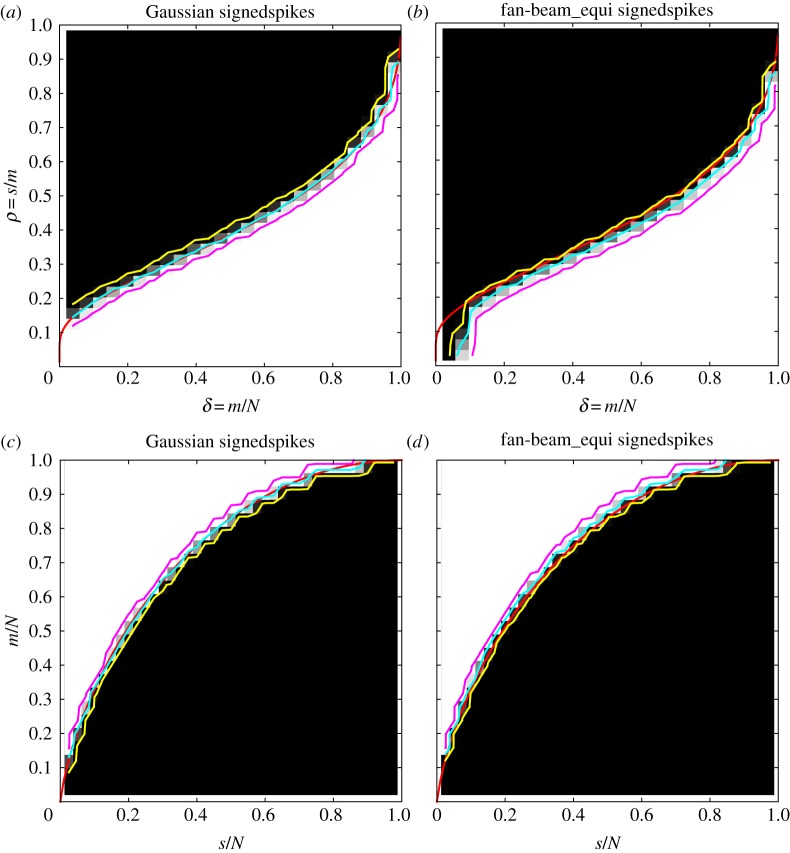
Phase diagrams for the signedspikes image class and P_1_ reconstruction. DT phase diagrams (*a*,*b*) and ALMT phase diagrams (*c*,*d*). Gaussian sensing matrices (*a*,*c*) and fan-beam CT system matrices (*b*,*d*). Theoretical phase-transition curves for Gaussian sensing matrices (red), empirical phase-transition curve at 50% contour line (cyan), and 5% and 95% contour lines (yellow and magenta).

We generate DT and ALMT phase diagrams as described in §2b for Gaussian and fan-beam CT sensing matrices (figure 2). At each sparsity and sampling level, the colour represents the empirical success rate, ranging from 0% (shown black) to 100% (shown white). Overlaid in cyan is the 50% contour line indicating the empirical transition curve, as well as in yellow and magenta the 5% and 95% contour lines to quantify the transition width. Further, in red is shown the theoretical phase-transition curve for the Gaussian sensing matrices.

We make the following observations. First, for the Gaussian sensing matrices, both the empirical DT and ALMT phase diagrams are in perfect agreement with the theoretical DT and ALMT phase-transition curves. This was to be expected but we include it here to verify that we can indeed reproduce the expected phase-transition curves using our software implementation. Second, and much more surprising, *the fan-beam CT phase diagrams are almost identical to the Gaussian case.* The single apparent difference is in the bottom left corner of the DT phase diagram, where the CT recovery region does not extend to the same level as the Gaussian case. The poor CT recovery performance here is easily explained: the two leftmost columns correspond to a single projection and two projections 180° apart, from which it is inherently difficult to produce an accurate reconstruction. Note that this issue is not apparent from the present ALMT phase diagram. Apart from this difference, the CT recovery performance is almost identical to the Gaussian case; in particular the transition is as sharp, as indicated by the 5% and 95% contour levels. Regarding the width of the transition, we have observed that smaller/larger images yield a wider/narrower transition region. This is in agreement with [28,31]. Furthermore, we observed that, if only a small number of repetitions is used, the transition generally appears wider. We found 100 repetitions to be sufficient for the transition width to stabilize. On very close inspection, the CT recovery region is slightly smaller than the Gaussian case, as seen by the lower cyan curve in the DT case and higher in the ALMT case.

Nevertheless, considering that the Gaussian sensing matrices form a near-optimal sampling strategy and that CT sampling matrices are highly structured, coherent and sparse, we find it extremely surprising to observe almost as good recoverability for CT.

#### (ii) Non-negative spikes by LP

Typically in CT, a non-negativity constraint can be employed as the imaged quantity, the linear attenuation coefficient, is non-negative, and hence the reconstruction problem LP is appropriate. For LP, we consider the natural non-negative version of the signedspikes class, which we call spikes, with the single change that values are sampled from the uniform distribution on [0,1] (see [22] for illustration).

**Figure 3.**
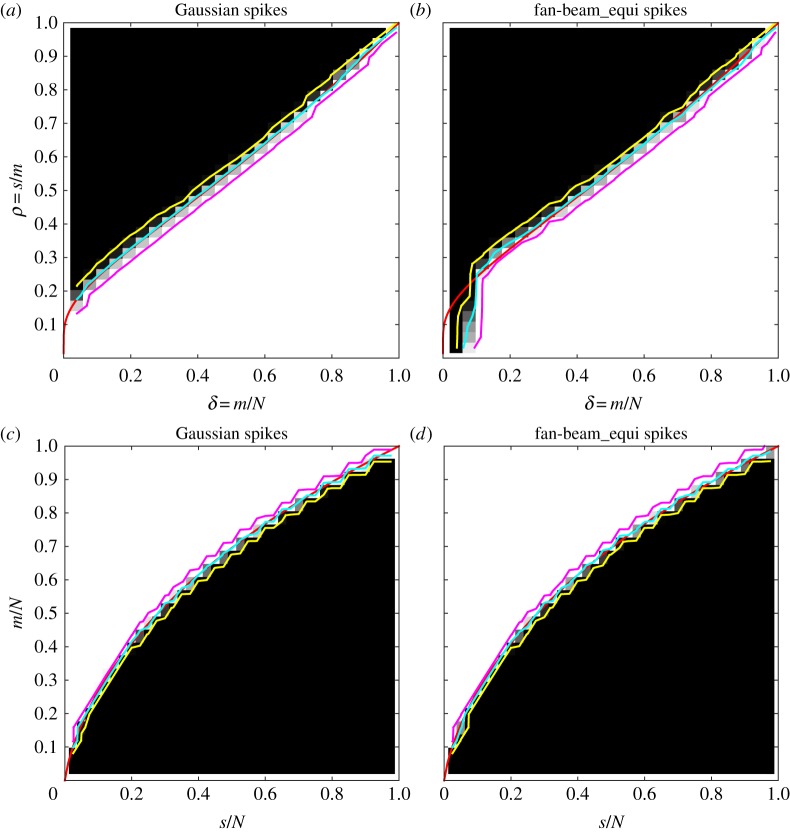
Phase diagrams for the non-negative spikes image class and LP reconstruction. DT phase diagrams (*a*,*b*) and ALMT phase diagrams (*c*,*d*). Gaussian sensing matrices (*a*,*c*) and fan-beam CT system matrices (*b*,*d*). Theoretical phase-transition curves for Gaussian sensing matrices (red), empirical phase-transition curve at 50% contour line (cyan), and 5% and 95% contour lines (yellow and magenta).

We construct again empirical DT and ALMT phase diagrams and display them in figure 3 together with the theoretical Gaussian-case phase-transition curves for LP. Also in this case, the CT phase diagrams are almost identical to the Gaussian case, in terms of both the empirical phase-transition curve and the width as indicated by the 5% and 95% contour lines. In fact, the similarity is even larger, as the cyan 50% contour in the CT case coincides with the theoretical transition curve, except at the bottom left corner of the DT phase diagram, as before caused by having only one or two CT projections. In accordance with the theoretical curves, we see that even fewer samples suffice for recovery in the non-negative case compared to before.

#### (iii) A structured image class

CS recovery guarantees, for example for the Gaussian sensing matrices state, that the sufficient number of samples depends on the signal only in terms of the signal sparsity. That is, signals with structure in the non-zero locations should not require a different number of samples for recovery than unstructured signals such as the spikes images. Does the same hold for CT? We will demonstrate that the answer is no. Owing to non-zero pixels selected at random in the spikes classes, there is no structure, i.e. correlation between neighbouring pixels. As an example of a class of sparse images with some structure in the non-zero locations, we use the 2-power class from [22]. This image class is based on a breast tissue model, but for our purpose here, it suffices to say some correlation has been introduced between neighbouring pixel values.

**Figure 4.**
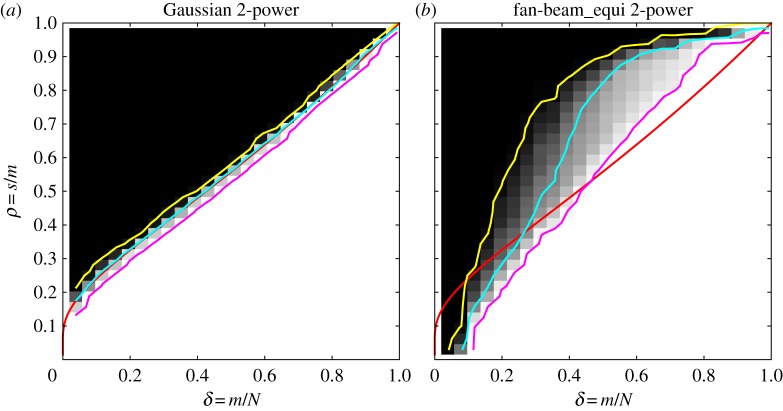
DT phase diagrams for the 2-power image class and LP reconstruction. Gaussian sensing matrices (*a*) and fan-beam CT system matrices (*b*). Theoretical phase-transition curves for Gaussian sensing matrices (red), empirical phase-transition curve at 50% contour line (cyan), and 5% and 95% contour lines (yellow and magenta).

Images from the 2-power class are non-negative, so we use LP for reconstruction, create DT phase diagrams (figure 4) and compare with the spikes-class DT phase diagrams in figure 3, omitting ALMT phase diagrams for brevity. As expected, our results verify that image structure does not matter for the Gaussian sensing matrices, as the DT phase diagram is identical to the spikes case. But, for the fan-beam CT case, the phase diagram has changed drastically; most notably the transition is now much smoother as indicated by the 5% and 95% contour lines. Also the empirical phase-transition curve (50% contour line) has moved away from the theoretical curve. We note that at low sampling (left part), the transition is lower, while at high sampling, it is higher, so recoverability can be both better and worse, depending on sampling level. The 95% contour line limits a region of almost full recovery, and this region is not much different from the spikes case.

The 2-power result for CT is in stark contrast to the Gaussian sensing matrix behaviour in figure 3. We conclude that, even though the spikes results suggest close resemblance of CT with the near-optimal CS case of Gaussian sensing matrices, CT is clearly more complex.

### (c) Gradient-domain sparsity

Sparsity in the image domain is interesting due to well-developed theory, in particular for Gaussian sensing matrices. For CT, it is more common to expect sparsity in the gradient domain, which has motivated the successful use of TV regularization. However, to the best of our knowledge, no phase-transition behaviour has been proved, not even for the Gaussian case. Here, we demonstrate empirically that for both Gaussian and CT sensing matrices similar sharp phase transitions can be observed.

For generating images sparse in the gradient domain, we use the image class from [23] alternating projection for (isotropic) TV, which we here refer to as altprojisotv. An image is generated in an iterative procedure of taking alternating projections onto the range of the gradient operator and thresholding the number of non-zeros in the image gradient to the desired sparsity level (see [23] for details and illustration).

**Figure 5.**
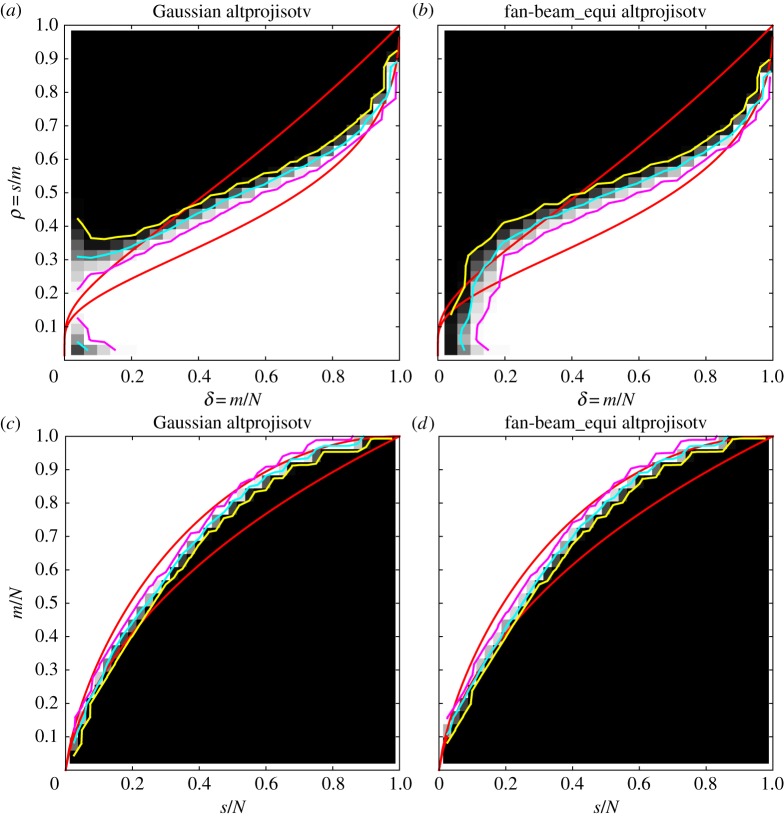
Phase diagrams for the altprojisotv image class and TV reconstruction. DT phase diagrams (*a*,*b*) and ALMT phase diagrams (*c*,*d*). Gaussian sensing matrices (*a*,*c*) and fan-beam CT system matrices (*b*,*d*). Theoretical phase-transition curves for P_1_ and LP reconstruction for Gaussian sensing matrices (red), empirical phase-transition curve at 50% contour line (cyan), and 5% and 95% contour lines (yellow and magenta).

Once again, we construct DT and ALMT phase diagrams (figure 5); this time with sparsity values referring to gradient-domain sparsity. We observe also in this case a sharp phase transition in both the DT and ALMT phase diagrams. In the lack of a theoretical reference curve for TV, we compare with the P_1_ and LP curves and find that transition takes place between the two curves.

An irregularity is observed in the bottom left corner of both DT phase diagrams. The explanation is that the altprojisotv procedure has difficulty in generating images which are extremely sparse in the gradient domain. In spite of the irregularity, we find that our empirical TV results convincingly demonstrate that sharp phase transition takes place also in the TV case, dividing the phase space into regimes of full and no recovery, and again that CT recoverability is similar to the Gaussian case.

### (d) Conclusion on Study A

We used phase-diagram analysis to compare fan-beam CT recoverability with near-optimal CS sampling using the Gaussian sensing matrices. For unstructured signed images with P_1_ and non-negative images with LP, we found almost identical phase-transition behaviour in terms of critical sampling level and width of the transition. We thereby demonstrated that empirically fan-beam CT in the average case performs close to the near-optimal. While recoverability by the Gaussian sensing matrices was unaffected by the introduction of structure in the non-zero pixels, fan-beam CT recoverability drastically changed to a much smoother transition. Interestingly, except for the lowest sampling range, the recovery region actually became larger, meaning that many images at a given sparsity level are recovered from fewer samples than the Gaussian sensing matrices' critical sampling level. In spite of the close resemblance on the unstructured images, this example demonstrates that fan-beam CT is fundamentally different from the Gaussian sensing matrices.

Also in the case of TV recoverability, we found almost identical behaviour of fan-beam CT and the Gaussian sensing matrices. In particular, in both cases, we saw a sharp phase transition, thus suggesting that the phase-transition phenomenon generalizes to TV. To our knowledge, no theoretical explanation of this observation has been given in the literature.

## 4. Study B. Is random sampling beneficial in CT?

As mentioned in the Introduction, random sampling is a near-optimal strategy and important in many recovery guarantees. Sampling in CT is normally done in a very structured manner and a natural contemplation is therefore whether the introduction of some form of randomness could lead to recovery guarantees for CT or improved recoverability compared to regular sampling. In this study, we use phase-diagram analysis to investigate whether CT sampling strategies involving randomness can improve the recoverability of sparse images, i.e. enable accurate reconstruction of images of a given sparsity from fewer measurements than regular equi-angular fan-beam CT.

### (a) Measurement matrices

Many forms of randomness can be conceived in CT sampling. In this work, we consider two straightforward ones. The first is a fan-beam geometry denoted fanbeam_rand in which the source angular positions are no longer equi-distant but sampled uniformly from [0,360°]. Second, we consider a set-up we denote random_rays of independent random rays through the image. Each ray is specified by two parameters: the angle of the ray with a fixed coordinate axis and the intersection of the ray with the orthogonal diameter of the disc-shaped image. The angle and intersection are sampled from uniform distributions on [0,180]° and [−*N*_side_/2,*N*_side_/2], respectively, where *N*_side_ is the diameter length and the image is assumed centred around the origin.

### (b) Image-domain sparsity

**Figure 6.**
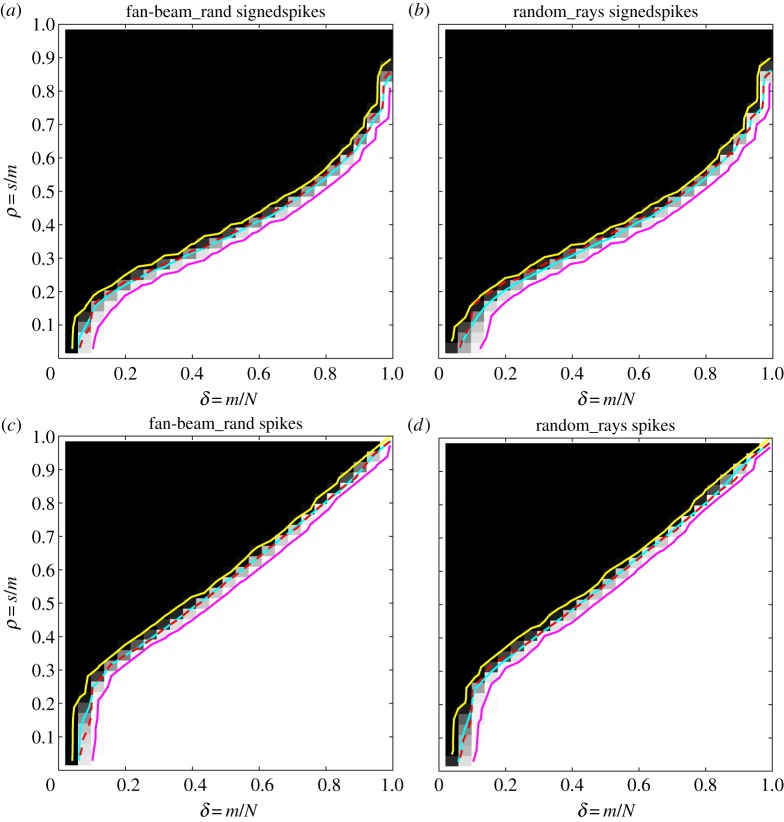
DT phase diagrams. Signedspikes image class and P_1_ reconstruction (*a*,*b*) and spikes image class and LP reconstruction (*c*,*d*). Fan-beam with random source positions (*a*,*c*) and random rays geometry (*b*,*d*). Empirical phase-transition curve for equi-angular fan-beam CT (dashed red), empirical phase-transition curve at 50% contour line (cyan), and 5% and 95% contour lines (yellow and magenta).

We create DT phase diagrams as in the previous section for the signedspikes class reconstructed by P_1_ and spikes reconstructed by LP (figure 6). ALMT phase diagrams are omitted for brevity. As the purpose of this study is to compare not with the Gaussian sensing matrices but with equi-angular fan-beam CT sampling, we do not show the theoretical phase-transition curves as in the previous section but instead, with the dashed red line, the empirical phase-transition curves for the equi-angular fan-beam CT geometry, which was shown in cyan in figures 2 and 3.

Compared to the equi-angular fan-beam case, we observe essentially no difference for the fanbeam_rand case: the empirical phase-transition curves follow the dashed red line closely in both signedspikes with P_1_ and spikes with LP phase diagrams. The random_rays set-up has very similar phase diagrams, but in the signedspikes case, the transition curve is slightly lower than in the equi-angular fan-beam case. In other words, on this set of image-domain sparsity test cases, randomness does not lead to improved recoverability, but rather to comparable or slightly reduced recoverability.

### (c) Gradient-domain sparsity

**Figure 7.**
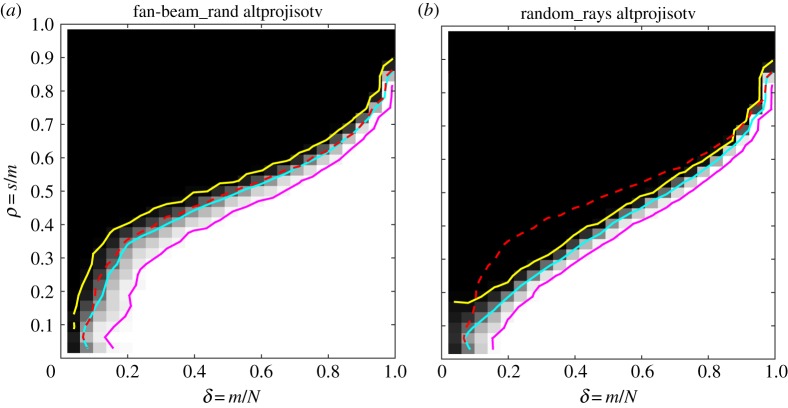
DT phase diagrams for the altprojisotv image class and TV reconstruction. Fan-beam with random source positions (*a*) and random rays geometry (*b*). Empirical phase-transition curve for equi-angular fan-beam CT (dashed red), empirical phase-transition curve at 50% contour line (cyan), and 5% and 95% contour lines (yellow and magenta).

For TV, we create phase diagrams for the altprojisotv class with both of the random-sampling CT set-ups (figure 7) and compare with the equi-angular fan-beam results in figure 5, indicated again by dashed red line. In both TV cases, we observe *worse* recoverability than for equi-angular fan-beam.

The fanbeam_rand set-up has a slightly lower empirical phase-transition curve and the transition is wider than for equi-angular fan-beam, as indicated by the larger distance between the 5% and 95% contour lines. This means that on average slightly more projections are needed to recover the same image and further that the critical sampling level sufficient for recovery is less well defined than for the equi-angular fan-beam case where the phase transition is sharper.

For random_rays, the transition curve is substantially lower, meaning that on average more measurements are needed for recovery of a same-sparsity image compared with the equi-angular fan-beam case. The largest difference is seen in the left half of the phase diagram, i.e. at fewer samples. One possible explanation of the reduced recoverability here is that, with relatively few and independent rays, the probability that some pixels are not intersected by any ray is relatively large. Thus, there is no information about such a pixel in the data, so the reconstructed value is solely determined by the regularizer. By contrast, in a fan-beam set-up with dense projection-view sampling as in our case, all pixels will be intersected by at least one ray from each projection view.

### (d) Conclusion on Study B

By use of phase-diagram analysis, we have compared two random-sampling strategies for CT with the more standard equi-angular fan-beam CT. The analysis revealed, in contrast to what might have been anticipated from the key role of randomness in CS, that random sampling does not improve recoverability in CT. On the contrary, in some cases, random sampling even leads to worse recoverability, most notably for the random_rays set-up.

## 5. Study C. Linking to realistic CT systems

In this section, we begin the task of linking the small-scale recovery results to realistic CT systems. What we are interested in is whether phase diagrams can be used to predict critical sampling levels as a function of sparsity in a realistic CT system. The studies presented should not be regarded as complete, and many issues for future research will be highlighted. Broadly speaking, the two main areas of concern are test phantom and optimization algorithm. A good test phantom presents a challenge. The small-scale phase-diagram results use phantom ensembles generated from a probabilistic model. While the results provide a sense of group recovery, a realization from any of the considered object models does not look like an actual object that would be CT scanned.

Which optimization algorithm to use is also an important question. For the small-scale studies, MOSEK is a convenient choice because a highly accurate solution can be computed reliably and reasonably fast. This means that whether or not an image is recoverable can be easily verified numerically. Optimization algorithms for large-scale CT systems cannot involve more expensive operations than matrix–vector products, at present, ruling out software packages such as MOSEK in favour of first-order methods that are inherently less accurate, in particular for large-scale problems, where in practice it is often necessary to truncate iteration early. As we will show, having less-accurate solutions makes it more difficult to decide whether an image is recoverable.

As large-scale studies are necessarily sparse, we cannot provide comprehensive empirical evidence of sufficient sampling but only a preliminary indication of how well phase-diagram analysis can predict sufficient sampling for SR for realistic CT systems. As we will show, even this is a complex task, for example due to complicated image structure and algorithmic issues, and we will point out several future directions to pursue.

Section 5a presents two phantoms generated for the present study to have different levels of realism with respect to an actual CT scanned object. Section 5b presents the first-order optimization algorithm we use for the large-scale recovery studies, while §5c illustrates some of the algorithmic and numerical challenges we face. Section 5d shows recovery results for the two phantoms as a function of number of CT projections and comparison with critical sampling levels predicted from small-scale phase diagrams.

### (a) Walnut test phantoms

In the present large-scale study, there are two links that need to be established to relate the small-scale phase-diagram analysis to realistic CT: the system size needs to be extrapolated up, in this case to *N*_side_=1024; and the results from the various probabilistic phantom models need to extend to realistic structure as seen in actual CT scan objects. We address both by designing two large-scale test phantoms with increasing realism from an actual CT scan of a walnut. The idea of scanning a walnut comes from [38].

In choosing a test phantom for image recovery studies, we aim for an image with gradient-domain sparsity to illustrate the effectiveness of TV in reducing the necessary number of samples for accurate image recovery. Yet, the phantom should also have features somewhat representative of what would be encountered in CT applications. Typical computer phantoms for CT image reconstruction testing, composed of simple geometric shapes of uniform grey levels, are unrealistically sparse in the gradient domain. Such phantoms would be helpful in extrapolation of small-scale phase-diagram analysis, but do not have much bearing in actual CT applications.

The basis of the test phantoms we generate is a cone-beam CT scan dataset of a walnut. The data consists of 1600 equi-angular 1024^2^-pixel projections acquired on a Zeiss Xradia 410 Versa micro-CT scanner operated at a 40 kV source voltage, 5 s exposure per projection, and 10.51 cm source-to-centre and 4.51 cm centre-to-detector distances. The central slice is reconstructed onto a 1024^2^-pixel image (pixel size 46.0773×10^−6^ m) from the corresponding rows of data using 500 iterations of a simultaneous iterative reconstruction technique (SIRT) algorithm.

**Figure 8.**
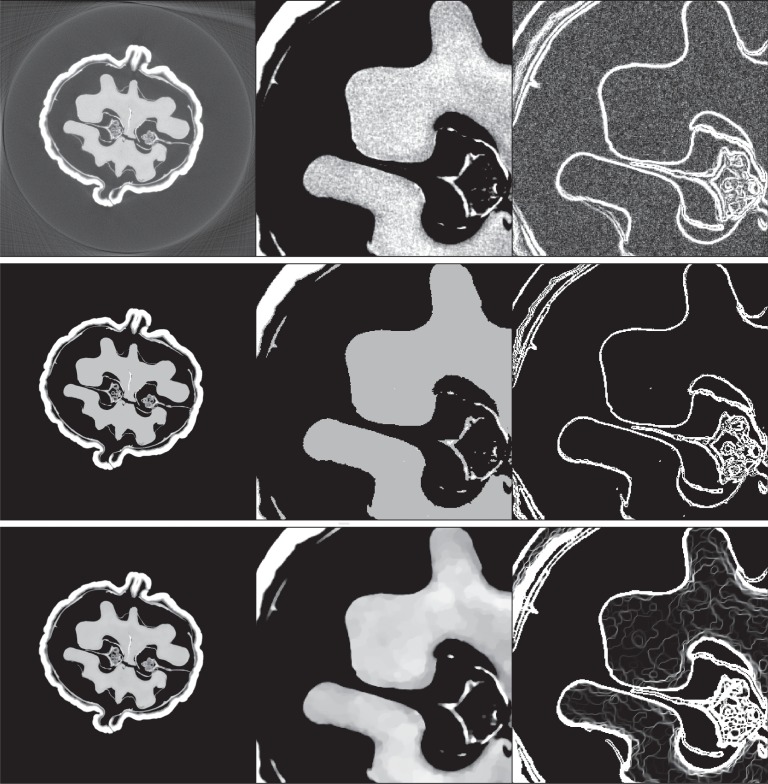
(Top row) Tomographic slice of a walnut, (middle row) structure phantom derived from the walnut slice image and (bottom row) texture phantom also derived from this image. The left column shows the whole image in the grey scale window of [0,0.5] cm^−1^, except for the original walnut image where it is [−0.1,0.5] cm^−1^. The middle column shows a blown-up region of interest in the narrower grey scale window [0.3,0.4] cm^−1^ in order to see the texture on the walnut meat. The right column illustrates the gradient-magnitude image in the grey scale window [0,0.01] cm^−1^, except for the original walnut image where it is [0,0.05] cm^−1^.

The first and simplest phantom, the *structure* phantom, is derived from the resulting image by equalizing the image grey-value histogram to seven discrete grey levels, including the background value of 0. The second and more complex phantom, the *texture* phantom, is derived from the walnut image by performing TV-denoising on the original walnut image after thresholding small background pixel values to zero. The two versions of the walnut phantoms including blow-ups and gradient-domain images are shown in figure 8 and gradient-domain sparsity values are given in table 1. The studies are idealized in that there is no data inconsistency; in actual CT, the projection data *b* will in general not be in the range of the projection operator *A*, and there is in this case no solution to the linear system *Ax*=*b*.

**Table 1.** Walnut test images with gradient-domain sparsity levels, number of projections at which recovery is observed, and DT and ALMT phase-diagram predictions of critical sampling levels. A reference point of full sampling is *N*_v_≥403 projections, where the system matrix has more rows than columns.

walnut image	gradient sparsity	recovered at	DT prediction	ALMT prediction
structure	45 074	68	69.3	71.7
texture	186 306	?	188.7	185.8

### (b) Large-scale first-order optimization algorithm

We consider large-scale solvers for problems P_1_ and TV. There has been much recent research on first-order algorithms [39,40], motivated by exactly the type of problem we face here. We require a solver that can handle the non-smoothness of P_1_ and TV, and which can be applied to large-scale systems such as CT, where the images can contain 10^6^ pixels in two dimensions or 10^9^ voxels in three dimensions and datasets of similar size. The CT system specifically presents another challenge in that the system matrix representing standard X-ray projection has poor conditioning [41]. An additional difficulty in solving P_1_ and TV, compared to the form (1.1), is in satisfying the equality constraint; achieving this constraint to numerical precision with present computational and algorithmic technology is not possible as far as we know. We present, here, our adaptation of the Chambolle–Pock (CP) primal-dual algorithm, which we have found to be effective for the CT system [42–44].

The algorithm used is essentially the same as the one developed in [44]. The CP algorithm instance is designed to solve the following optimization problem:
5.1
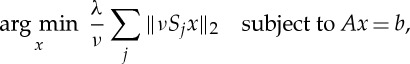

where equation (5.1) becomes P_1_ and TV when the sparsifying operator is *S*_*j*_=*I*_*j*_ and *S*_*j*_=*D*_*j*_, respectively; *I*_*j*_ is an image where the *j*th pixel is one and all other pixels are zero; *ν* is a constant which balances the operator norms

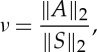

where *S* is a matrix of *S*_*j*_ for all *j*; and the parameter λ, which does not affect the solution of equation (5.1), is used to improve numerical convergence. The parameter λ is tuned empirically. The corresponding algorithm for solving equation (5.1) is shown in pseudo-code form in algorithm 1.


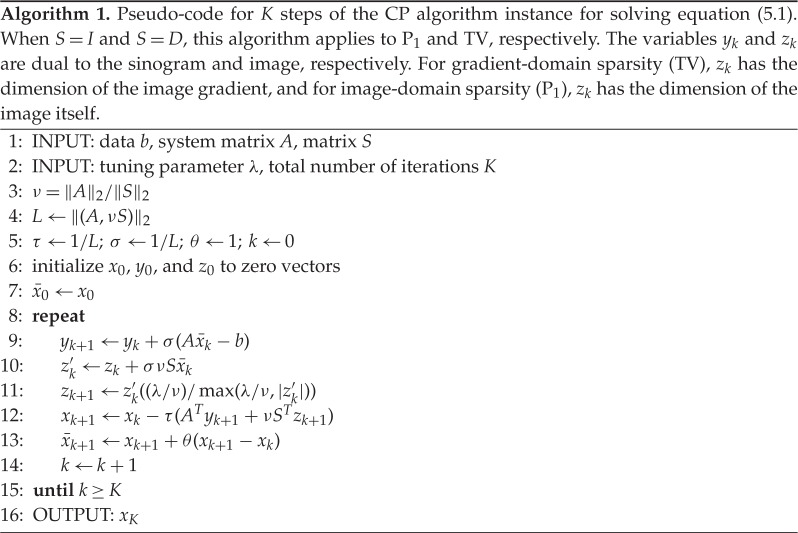


Considering that the phantom-recovery studies for which we want to use algorithm 1 involve multiple runs over different system matrices *A* corresponding to CT sampling with different numbers of projections, we found it most practicable to obtain results for fixed iteration number *K* and tuning parameter λ. The computational time for performing the expensive operations *Ax* and *A*^T^*x* makes consideration of a prescribed stopping criterion difficult. For the *N*_side_=1024 system of interest, these time-limiting operations take 1 s for our GPU-accelerated projection codes. A fixed stopping criterion entails variable numbers of iterations, and we have observed that for algorithm 1 the number of iterations can vary from 1000 to over 100 000 for a convergence criterion of interest. In terms of computational time, this range translates to 20 min to well over a day. As a result, a study may not be completed in a reasonable amount of time; thus, we fix *K* and λ for our phantom-recovery study.

Because large-scale first-order optimization algorithms are seeing many new developments at present, it is likely that there either exists or will be a better alternative to algorithm 1. In fact, we invite the interested reader to find such an alternative, which can have an important impact on CT imaging! For example, as will be seen shortly, algorithm 1 has limited success for phantom recovery studies for P_1_ on systems of realistic size.

### (c) Algorithm issues

We demonstrate first some of the challenges in carrying out large-scale recovery studies by applying algorithm 1 to a medium-scale problem using an *N*_side_=128 version of the structure walnut phantom. The phantom has a gradient-domain sparsity of 1826 and a pixel sparsity of 2664 out of a total of 11 620 pixels. We do a recovery study by studying the reconstruction root-mean-square error (RMSE) as a function of the number of projections. We discuss in detail specific issues of the sampling recovery study for the purpose of understanding the large-scale results.

#### (i) The tuning parameter λ and convergence

**Figure 9.**
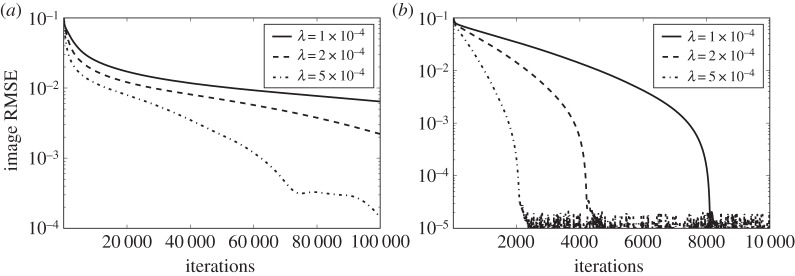
Image RMSE curves resulting from algorithm 1 run with different values of λ for *N*_v_=21 and data generated from the *N*_side_=128 version of the structure walnut phantom. Results for P_1_ and TV are shown on (*a*) and (*b*), respectively.

The tuning parameter λ does not affect the solution of P_1_ or TV, but it can have a large impact on convergence. To illustrate this, we show results of single runs for *N*_side_=128 and *N*_v_=21 for both P_1_ and TV in figure 9. The value *N*_v_=21 is chosen because it is the smallest number of views for which accurate recovery is obtained for both P_1_ and TV. Note that we are showing results for *K*=100 000 iterations for P_1_, while only *K*=10 000 for TV. It is clear that convergence rates change significantly with λ, and consequently recovery curves will be affected by λ. While λ is specific to algorithm 1, optimization algorithms generally entail parameters with large effects on convergence rate.

**Figure 10.**
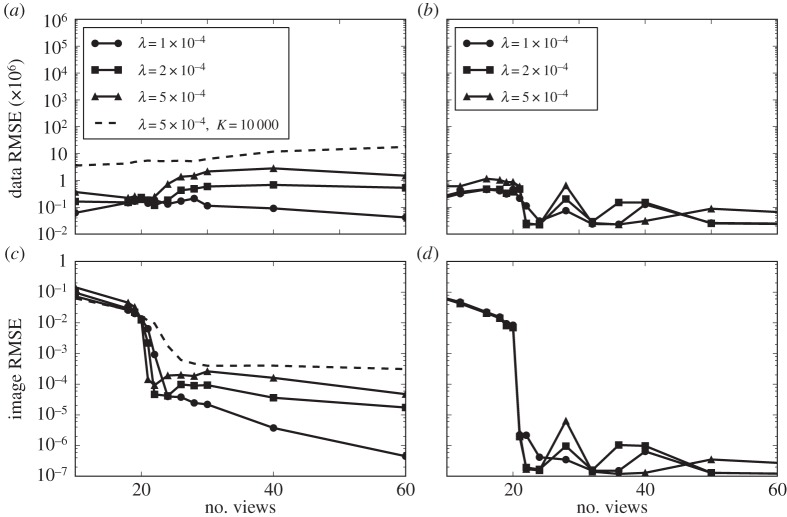
Image and data RMSE plots for the *N*_side_=128 version of the structure walnut phantom using algorithm 1 with different values of λ. The results for P_1_ (*a*,*c*) are obtained for *K*=10^5^ iterations except for the indicated curve for *K*=10^4^. The results for TV (*b*,*d*) are obtained for *K*=10^4^ iterations.

The impact on recovery curves is seen in figure 10, where we compare recovery curves obtained at different λ for P_1_ (*K*=100 000 iterations) and TV (*K*=10 000 iterations). While overall the recovery curves are similar, some differences appear, in particular near the jump in error for P_1_. This can complicate the accurate estimation of the jump location. Overall, in this case, the lowest image RMSE is obtained for λ=5×10^−4^. For the large-scale system *N*_side_=1024, we have found the value of λ=1×10^−4^ to be useful for P_1_ and TV, and for different values of *N*_v_ and *N*_side_. One could envision a strategy where algorithm 1 is run with a small set of λ values and the lowest image RMSE at iteration *K* is taken for the recovery plot. In the large-scale results presented shortly, we found this to be unnecessary, and λ is simply fixed at 1×10^−4^.

#### (ii) Recovery plots and difficulty with P_1_

The phantom recovery plots for P_1_ and TV in figure 10 both show the distinct jump in RMSE at a certain number of projections, at which the image is recovered. We recognize this from the small-scale *N*_side_=64 studies in [22]. The price of using fixed *K*, however, is that convergence results across projection numbers are not uniform, as the data discrepancy varies with view number.

Furthermore, the recovery curve can be severely affected by poor convergence. If instead of *K*=100 000 we only take *K*=10 000 as in the TV case, the remaining recovery curve in figure 10 is obtained. The previously abrupt change in error is considerably smoothed and shifted to a different number of views.

The issue of convergence, here, is ubiquitous in iterative image reconstruction for CT and it can be traced to the use of matched projection, *A*, and back-projection, *A*^T^, where it is well known in the CT community that matched projector/back-projector pairs can lead to Moiré artefacts that decay extremely slowly [45]. As a result, many iterative algorithms in CT employ a different back-projection matrix *B*≠*A*^T^ [46]. For our purpose, we must use the matched pair, in order to solve a well-defined optimization problem. For the larger system, sufficient iteration for P_1_ lies out of reach with algorithm 1 and we focus only on phantom recovery for TV.

### (d) Large-scale recovery results

#### (i) Predicting sufficient sampling from phase diagrams

We will use the phase diagrams from Study A to predict critical sampling levels for large-scale TV reconstruction. We found in [22] that the ALMT phase diagram of a given image class remains unchanged at image resolutions *N*_side_=32, 64 and 128, i.e. is independent of resolution. We assume this holds also for the DT phase diagram, and we use the DT and ALMT phase diagrams from figure 5 (which are for *N*_side_=64) to predict critical sampling levels for the two walnut images at *N*_side_=1024.

**Figure 11.**
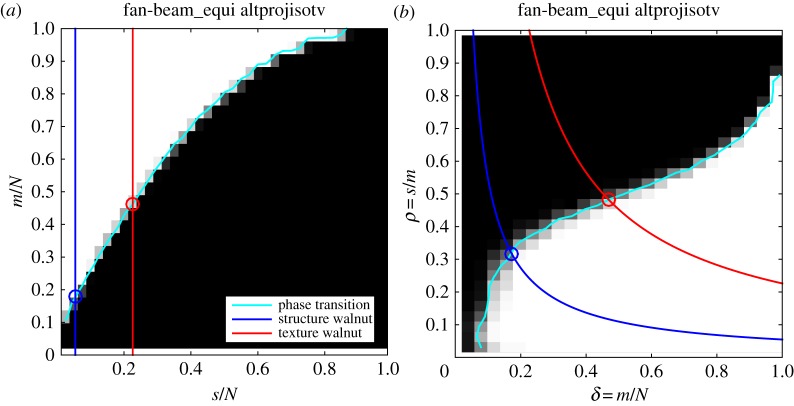
Prediction of critical sampling for TV and walnut phantoms by ALMT (*a*) and DT (*b*) phase diagrams.

We illustrate in figure 11 how to determine critical sampling levels given a sparsity level. The number of pixels inside the disc is 823 592 and the gradient sparsity levels of the structure and texture walnut images are given in table 1. In the ALMT phase diagram, we can trace vertical lines at each *s*/*N* value and find the intersections (indicated by circles) with the empirical phase-transition curve, which gives the predicted critical *m*/*N* values. By multiplication of *N* and division by the number of rays in a single projection, i.e. 2048, we get the critical number of projections (table 1).

To do the same in the DT phase diagram, we combine *δ*=*m*/*N* and *ρ*=*s*/*m* into *ρ*=(*s*/*N*)(1/*δ*), i.e. a fixed sparsity *s* traces out a hyperbola on *δ*∈(0,1). For the hyperbola of each walnut image, we find the intersection point (*m*/*N*,*s*/*m*) with the empirical phase-transition curve. Up to the accuracy of reading off the figure, the two components lead to identical critical values of *m*, from which we find the critical number of projections for each walnut image (table 1).

We note that the larger number of gradient non-zeros in the texture walnut image leads to prediction of a higher critical sampling level. Similar plots for image-domain sparsity could be constructed based on figures 2 and 3 and the fixed-sparsity curves would then reflect that the walnut images have more non-zeros in the pixel domain than in the gradient domain, yielding higher predicted critical sampling levels for P_1_/LP than for TV.

#### (ii) Recovery of the large-scale walnut phantoms

**Figure 12.**
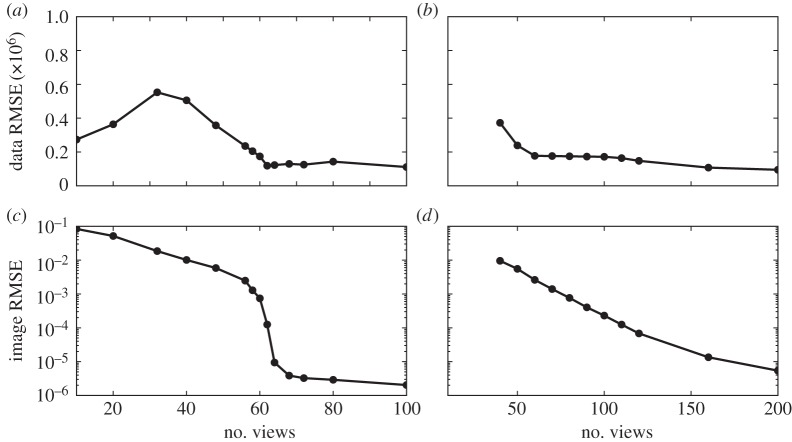
Image and data RMSE plots for the *N*_side_=1024 structure (*a*,*c*) and texture (*b*,*d*) walnut phantom using algorithm 1 with λ=10^−4^. The results are obtained at *K*=10^4^ iterations.

We employ algorithm 1 to solve TV on the large-scale *N*_side_=1024 CT system for the structure and texture walnut phantoms. The resulting recovery plots are shown in figure 12. For the structure walnut, we observe an abrupt change in image RMSE with *N*_v_=68 yielding accurate recovery, as decided by the first point where there is essentially no further decrease in RMSE. The predicted critical sampling levels from DT and ALMT phase-diagram analysis are only slightly higher at *N*_v_=69.3 and *N*_v_=71.7, respectively (cf. table 1). This result is rather remarkable in that the extrapolation is extended quite far from the size of the original phase-diagram analysis. Also, the structure phantom is clearly different from any expected realization of any of the studied probabilistic phantoms models.

The recovery curve for the texture phantom, on the other hand, does not exhibit an abrupt change in reconstruction error, rather a gradual improvement all the way up to 200 projections. We therefore cannot point to a specific critical sampling level.

#### (iii) Reconstructed images for the structure and texture phantoms

**Figure 13.**
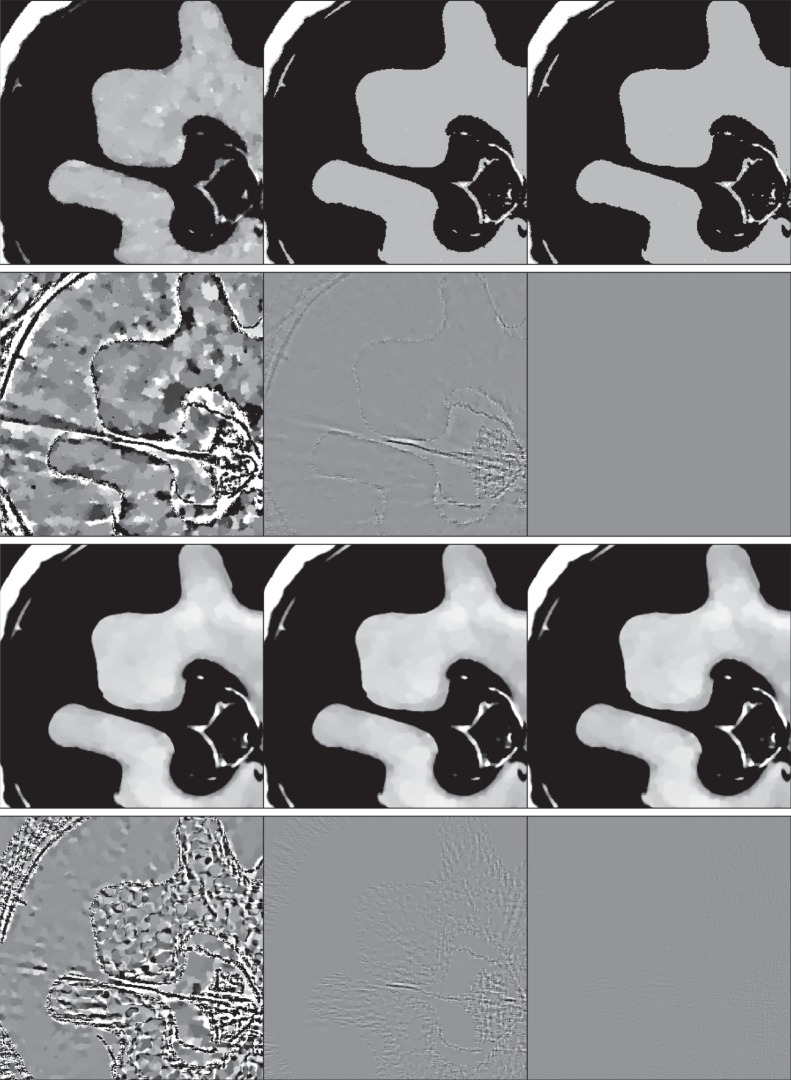
First row: reconstructed images from data generated by the structure walnut with 40 (left), 60 (middle) and 68 (right) projection views (grey scale window [0.3,0.4] cm^−1^). Second row: same as first row except the structure walnut image is subtracted from the reconstructed images (grey scale window [−0.01,0.01] cm^−1^). Third row: reconstructed images from data generated by the texture walnut with 80 (left), 120 (middle) and 160 (right) projection views (grey scale window [0.3,0.4] cm^−1^). Fourth row: same as third row except the texture walnut image is subtracted from the reconstructed images (grey scale window [−0.001,0.001] cm^−1^).

It is illuminating to inspect some of the reconstructed images in figure 13, which correspond to the plots in figure 12. The second and third reconstructions for the structure phantom straddle the sharp transition in the corresponding image RMSE curve, and it can be seen clearly in the difference image that the result for *N*_v_=60 is not recovered, while that for *N*_v_=68 is much closer to the test phantom. We point out, however, that the difference images are displayed in a narrow 4% grey scale window and visually the *N*_v_=60 image appears the same as the structure phantom. That the discrepancies between reconstruction and phantom are so small emphasizes the challenge for the large-scale optimization algorithms; for actual application where images are presented for visual inspection, such accurate solution to equation (1.1) would not be necessary. The results for the texture phantom are also quite interesting in that we see the reconstructed image is visually accurate for as few views as *N*_v_=80. That there is no sharp recovery transition for the texture phantom is probably due to the fact that the object variations occur on two scales: the jumps of the structure borders, and the splotches of the walnut meat texture. It also cannot be ruled out that a sharper recovery transition will occur if the accuracy of the computed solutions is improved even further.

### (e) Conclusion on Study C

In this study, we have taken first steps towards phase-diagram analysis for prediction of critical sampling levels for realistic CT systems. Both test phantom design and accurate large-scale optimization are more difficult than for small-scale studies, and we have demonstrated how phantom appearance as well as parameters and convergence of the algorithm can affect recovery studies. For the simplest, and piecewise constant, structure walnut phantom, we found the critical sampling level to be predicted very well by phase-diagram analysis. The situation for the texture walnut phantom was more complex, which motivates further and more extensive large-scale studies, including of the influence of texture on recovery and possibly a different definition of image recovery itself.

## 6. Conclusion

We have presented a systematic framework of phase-diagram analysis from CS for analysing the undersampling potential of SR in X-ray CT. In three, quite different, studies, we have demonstrated the potential of phase-diagram analysis. We saw that, under certain conditions, X-ray CT in terms of recoverability performs comparably with a near-optimal CS sampling strategy of Gaussian sensing matrices; that random sampling in X-ray CT in terms of recoverability does not perform better, and in some cases is worse, than a regular fan-beam sampling set-up; and that, at least in a simple case, the critical sampling level for a large-scale X-ray CT system can be predicted. An interesting future direction is to address the question: Can the observed phase-transition behaviour in X-ray CT be theoretically explained, in particular the high degree of similarity with the Gaussian sensing matrix case?

## Data accessibility

Supporting data and code are available in Dryad Digital Repository at http://dx.doi.org/10.5061/dryad.3jg57.
